# AMCSMMA: Predicting Small Molecule–miRNA Potential Associations Based on Accurate Matrix Completion

**DOI:** 10.3390/cells12081123

**Published:** 2023-04-10

**Authors:** Shudong Wang, Chuanru Ren, Yulin Zhang, Shanchen Pang, Sibo Qiao, Wenhao Wu, Boyang Lin

**Affiliations:** 1 College of Computer Science and Technology, Qingdao Institute of Software, China University of Petroleum, Qingdao 266580, China; 2 College of Mathematics and Systems Science, Shandong University of Science and Technology, Qingdao 266580, China

**Keywords:** MicroRNA, small molecule, association prediction, truncated nuclear norm regularization, matrix completion

## Abstract

Exploring potential associations between small molecule drugs (SMs) and microRNAs (miRNAs) is significant for drug development and disease treatment. Since biological experiments are expensive and time-consuming, we propose a computational model based on accurate matrix completion for predicting potential SM–miRNA associations (AMCSMMA). Initially, a heterogeneous SM–miRNA network is constructed, and its adjacency matrix is taken as the target matrix. An optimization framework is then proposed to recover the target matrix with the missing values by minimizing its truncated nuclear norm, an accurate, robust, and efficient approximation to the rank function. Finally, we design an effective two-step iterative algorithm to solve the optimization problem and obtain the prediction scores. After determining the optimal parameters, we conduct four kinds of cross-validation experiments based on two datasets, and the results demonstrate that AMCSMMA is superior to the state-of-the-art methods. In addition, we implement another validation experiment, in which more evaluation metrics in addition to the AUC are introduced and finally achieve great results. In two types of case studies, a large number of SM–miRNA pairs with high predictive scores are confirmed by the published experimental literature. In summary, AMCSMMA has superior performance in predicting potential SM–miRNA associations, which can provide guidance for biological experiments and accelerate the discovery of new SM–miRNA associations.

## 1. Introduction

MicroRNAs (miRNAs) are a class of single-stranded noncoding RNA molecules containing 17–24 nucleotides [1,2,3]. The first miRNA, lin-4, and the first mammalian miRNA, let-7, were found in the 1990s [4,5]. With these two significant discoveries, a wave of genomic research took place, resulting in the discovery of a large number of miRNAs in many organisms [6,7]. At the same time, it has become increasingly evident to researchers that miRNAs are involved in complex and diverse life processes. Specifically, miRNAs can bind to complementary target mRNAs, resulting in mRNA translational inhibition or degradation, which means that miRNAs have a significant impact on cell differentiation, proliferation, and apoptosis [1]. In addition, miRNAs play essential roles in various cellular activities, including immune responses and neurotransmitter synthesis [8,9]. More significantly, miRNAs participate in tumorigenesis and host–pathogen interactions [10,11,12,13]. For instance, Liu et al. [14] identified that the abnormal expression of miR-181c is involved in the pathogenesis of glioblastoma. Therefore, restoring the expression level of miR-181c in glioblastoma cancer cells can effectively treat the disease, which also provides new insight for the clinical treatment of many refractory diseases, including cancer.

Indeed, as a low-molecular-weight compound, small molecule (SM) drugs have been demonstrated to target dysregulated miRNAs and modulate their expression [15,16,17]. For instance, SPC3649, the first miRNA-targeted drug administered in human clinical trials, successfully inhibits the expression of miR-122, which is required for hepatitis C virus replication [18]. Consequently, utilizing miRNAs as diagnostic and therapeutic targets has become a promising pathway in drug development and disease treatment. Since developing new SMs is time-consuming and expensive, it is extremely difficult to develop specific SMs for each dysregulated miRNA. Therefore, researchers can look into utilizing existing SMs to target and modulate a wider variety of miRNAs [19]. Furthermore, determining the potential associations between the known SMs and miRNAs through biological experiments is of great significance and urgency.

Given the abundance of existing SMs and miRNAs, it is critical to pre-screen out SM–miRNA samples with high association probabilities for complex and expensive biological experiments. The proposed predictive approaches may be divided into network inference-based models and matrix-completion-based models. For the first kind of model, Guan et al. [20] proposed a model called Graphlet Interaction-based Inference for Small Molecule–miRNA Association Prediction (GISMMA). Based on the integrated SM/miRNA similarity (the widely used SM/miRNA similarities include the side-effect-based SM similarity, the chemical-structure-based SM similarity, the functional consistency-based SM similarity, the indication phenotype-based SM similarity, the gene functional consistency-based miRNA similarity, and the disease-phenotype-based miRNA similarity), they first constructed the SM/miRNA similarity network. Then, a specific SM–miRNA association score was calculated by counting the number of graphlet interactions throughout the SM/miRNA similarity network. Furthermore, Li et al. [21] developed the Small Molecule–miRNA Network-Based Inference (SMiR-NBI) predictive model. In a constructed SM–miRNA heterogeneous network, a given SM node evenly distributes the obtained initial resources to the miRNA nodes regulated by it. Following this, these miRNA nodes immediately distribute the obtained resources to the SM nodes adjacent to them. As the resources are continuously propagated through the network, the resource allocation of all nodes eventually stabilizes. The final resource fraction of the miRNA nodes reflects the possibility of being regulated by the given SM. Notably, the model is incapable of predicting miRNAs or SMs that are potentially associated with new SMs or miRNAs. Additionally, Qu et al. [22] proposed the Triple Layer Heterogeneous Network-based Small Molecule–miRNA Association (TLHNSMMA) predictive model. They first constructed an SM–miRNA-disease triple layer heterogeneous network.  An iterative update algorithm was then applied to obtain the association scores of all SM–miRNA pairs. Benefiting from the introduction of additional information, the model demonstrated excellent prediction accuracy. However, it is likewise not applicable to predict miRNAs or SMs that are potentially associated with new SMs or miRNAs. In view of the unreliability of all the aforementioned methods due to the presence of noise data, Yin et al. [23] developed a new computational method called Sparse Learning and Heterogeneous Graph Inference for Small Molecule–miRNA Associations (SLHGISMMA) prediction. They first decomposed the SM–miRNA association matrix into two parts, in which the first part is a linear combination of the original association matrix and a low-rank matrix, and the second part is a sparse noise matrix. After eliminating the noise matrix, they integrated the SM/miRNA similarity information and the information in the reacquired association matrix into a heterogeneous graph. Finally, the association scores were obtained by implementing a heterogeneous graph inference algorithm. The drawback of SLHGISMMA is that it cannot restrict the prediction scores in [0, 1], which reduces the interpretability and accuracy of the association scores.

Additionally, several matrix-completion-based heuristic algorithms are likewise applied in predicting potential SM–miRNA associations. Inspired by the traditional CMF [24] method, Wang et al. [25] developed a model called Dual-Network Collaborative Matrix Factorization (DCMF) for predicting small molecule–miRNA associations. They first preprocessed the SM–miRNA association matrix utilizing the Weighted K-Nearest Known Neighbors (WKNKN) method. In addition to the Tikhonov regularization term, they incorporated two new regularization terms in the optimization framework of the traditional matrix factorization model. After solving the optimization problem, they calculated the matmul product of the two low-rank feature matrices as the completed matrix, and the completed values were considered as the association scores. Moreover, a model named Predicting Potential Small Molecule–miRNA Associations based on Bounded Nuclear Norm Regularization (BNNRSMMA) was developed by Chen et al. [26]. They recovered the target matrix with the missing values by minimizing its nuclear norm. Although BNNRSMMA restricts the completed value in [0, 1], it may obtain a highly biased solution as the nuclear norm may not be the optimal convex approximation to the rank function, and the prediction accuracy cannot be guaranteed. Considering that the rank of the result matrix is non-adjustable, this decreases the adaptability of BNNRSMMA on different datasets. The main innovative points and limitations of the above models are shown in Table 1.

**Figure 1:**
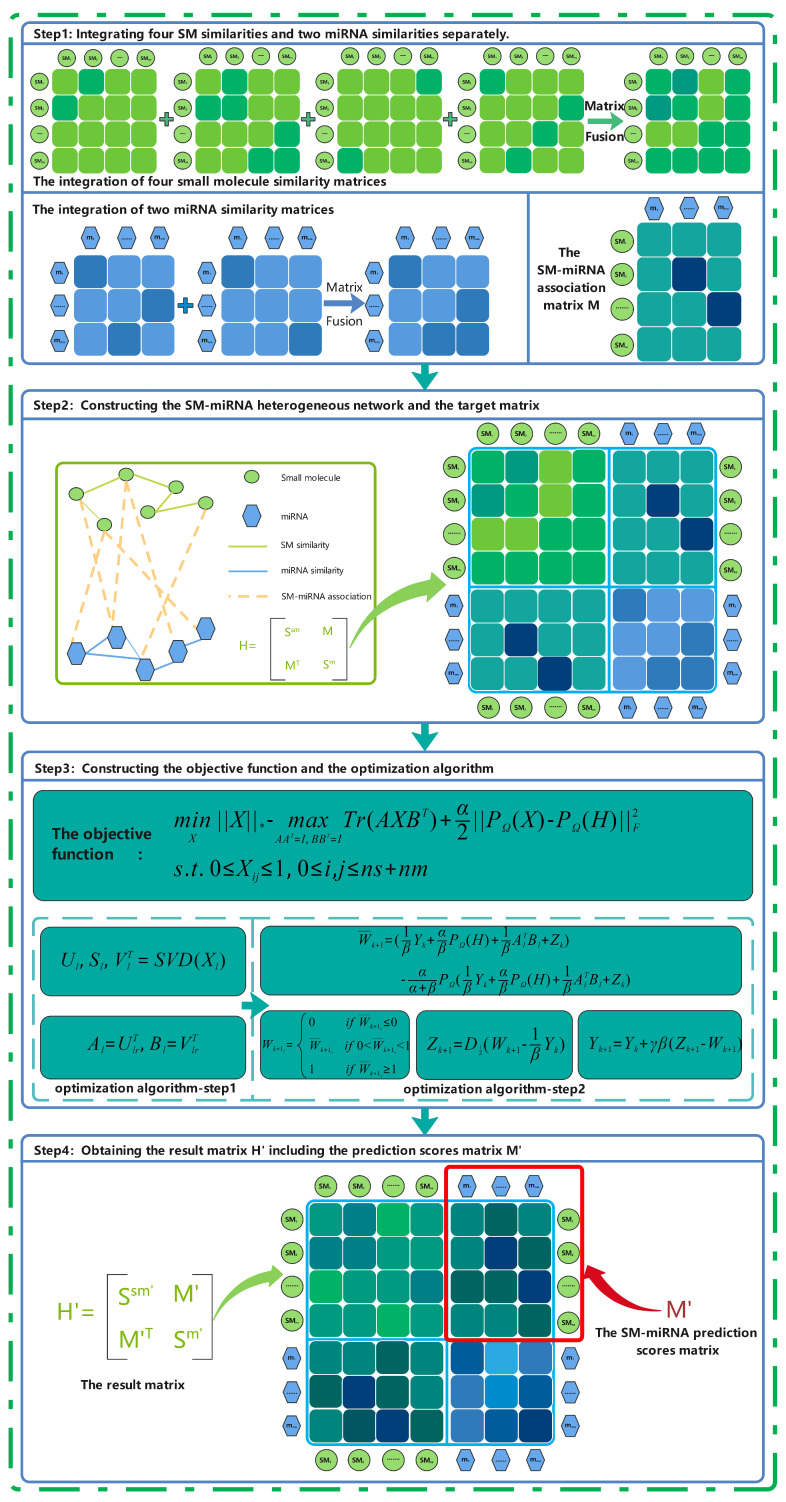
The framework of AMCSMMA. (**1**) Integrating different biological data similarities. (**2**) Constructing the SM–miRNA heterogeneous network and the target matrix. (**3**) Constructing the objective function and the optimization algorithm. (**4**) Obtaining the prediction score matrix through matrix division.

Considering that the previous models have some limitations, we develop a more accurate predictive model called AMCSMMA, which overcomes the insufficiencies listed in Table 1, and its framework is shown in Figure 1. In Validation Experiment A, the AUC scores achieve the best results with values ranging from 0.9974 to 0.9981 when parameter r≤13, and we finally set r∈{1,2,3} to reduce the computational complexity. Additionally, we design Validation Experiment B in which the values of the AUC, Precision, Recall, F1 Score, Accuracy, and MCC are all above 0.97. Moreover, we conduct four types of cross-validation (CV) experiments based on Dataset 1 (Dataset 2). As a result, the AUC values of AMCSMMA are 0.9910 ± 0.0004 (0.8768 ± 0.0039), 0.9923 (0.8861), 0.9898 (0.8880), and 0.8222 (0.7232) under five-fold CV, Global Leave-One-Out CV (LOOCV), miRNA-Fixed Local LOOCV, and SM-Fixed Local LOOCV, respectively, which are a significant improvement compared with previous models. In the first type of case study, 9 (33) among the top 20 (100) associations predicted by AMCSMMA are confirmed by the published experimental literature. In the second type of case study to the SMs 5-FU and 5-Aza-2’-deoxycytidine, 20 (34) and 16 (26) among the top 20 (50) associations are, respectively, verified by published references. In conclusion, AMCSMMA demonstrates superior accuracy and reliability in predicting potential SM–miRNA associations. It can be used for screening SM–miRNA samples with high association probabilities for complex biological experiments, thus significantly reducing the time and financial cost of discovering new SM–miRNA associations. This paper’s significant contributions are summarized as follows:

1. We integrate a variety of SM/miRNA similarities and consider the adjacency matrix of the constructed SM–miRNA heterogeneous network as the target matrix, which can not only effectively utilize the integrated similarity to improve the prediction accuracy but also enhance its information content as the iteration progresses.
2. We utilize the truncated nuclear norm regularization as the strategy to approximate the rank function, which not only achieves the rank minimization more accurately, robustly, and efficiently but also increases the adaptability to different datasets.
3. We design an effective two-step iterative scheme to solve the optimization problem. In order to solve the convex sub-problem in the second step, we introduce the Alternating Directional Multiplier Method (ADMM).

## 2. Materials and Methods

### 2.1. SM–miRNA Associations

In this study, we obtained 664 known SM–miRNA associations from the SM2miR v1.0 [27] database. Then, we collected 831 SMs from the SM2miR v1.0 [27], PubChem [28], and DrugBank [29] databases, as well as 541 human-related miRNAs from the HMDD [30], miR2Disease [31], PhenomiR [32], and SM2miR v1.0 [27] databases. The first dataset (Dataset 1) was constructed from all the data described above, which contained 831 SMs, 541 miRNAs, and 664 confirmed SM–miRNA associations.

The second dataset (Dataset 2) was then constructed by removing SMs and miRNAs without confirmed associations in Dataset 1. It contained 39 SMs, 286 miRNAs, and 664 identical known associations as Dataset 1. Moreover, we constructed a novel independent dataset (Dataset 3) that contained the identical 831 SMs and 541 miRNAs as Dataset 1 but with 132 additional known associations collected from the latest experimental literature (the complete information can be found on our Github page or Supplementary File).

To represent associations between SMs and miRNAs more directly, we constructed an association matrix $M\in R^{ns\times nm}$ in each dataset, where ns and nm, respectively, represent the number of SMs and miRNAs in the dataset. Specifically, each row of *M* represents a specific SM, and each column of *M* represents a specific miRNA. The (*i*,*j*)-th element of the association matrix, $m_{ij}$, is set to 1 if SM${}_{i}$ is associated with miRNA${}_{j}$, otherwise it is set to 0. Table 2 shows the complete data information for these three datasets.

### 2.2. Integrated SM Similarity

Referring to the previous work of Lv et al. [33], we introduce four kinds of widely used SM similarities: the side-effect-based SM similarity [34], the chemical-structure-based SM similarity [35], the functional consistency-based SM similarity [36], and the indication phenotype-based SM similarity [34].

- The side-effect-based SM similarity was calculated according to the Jaccard score based on the number of shared side effects between two SMs. The SM-related side effects were extracted from the SIDER [37] database.
- The chemical-structure-based SM similarity was calculated by analyzing the maximal common sub-graphs between the chemical structure graphs of two SMs.
- The indication phenotype-based SM similarity was calculated according to the similarity between MeSH [38] terms of diseases associated with SMs. The disease information related to SMs was extracted from the DrugBank [29] database.
- The functional consistency-based SM similarity was calculated based on the functional association between the target gene sets of SMs. The target gene information of SMs was extracted from the DrugBank [29] and TTD [39] databases.

We constructed four SM similarity matrices of dimension ns×ns (represented by $S_{S}^{sm}$, $S_{C}^{sm}$, $S_{F}^{sm}$, and $S_{P}^{sm}$) where each row and its corresponding column represent a specific SM. The element in the *i*-th row and *j*-th column denotes the similarity score between SM${}_{i}$ and SM${}_{j}$. To minimize the bias of a single similarity measure, we integrated these similarity matrices utilizing the weighted averaging strategy as follows:

(1) $$\begin{array}{cc} S^{sm} & =\frac{\alpha_{1}S_{S}^{sm}+\alpha_{2}S_{C}^{sm}+\alpha_{3}S_{F}^{sm}+\alpha_{4}S_{P}^{sm}}{\sum_{i=1}^{4}\alpha_{i}} \end{array}$$

where $S^{sm}\in R^{ns\times ns}$ indicates the integrated SM similarity matrix, and $\alpha_{i}$ denotes the weight of the *i*-th SM similarity matrix, which is set to 1.

### 2.3. Integrated miRNA Similarity

Similarly, we introduce two types of miRNA similarities: the gene functional consistency-based miRNA similarity [36] and the disease-phenotype-based miRNA similarity [34].

1. The gene functional consistency-based miRNA similarity was calculated based on the functional identity between target gene sets of miRNAs.
2. The disease-phenotype-based miRNA similarity was calculated according to the Jaccard score based on the number of shared diseases between two miRNAs. The miRNA-related diseases were extracted from three databases: HMDD [30], miR2Disease [31], and PhenomiR [32].

We constructed two miRNA similarity matrices of dimension nm×nm (represented by $S_{G}^{m}$, and $S_{D}^{m}$), in which each row and its corresponding column represent a specific miRNA. The (*i*,*j*)-th element denotes the similarity score between miRNA${}_{i}$ and miRNA${}_{j}$. The integrated similarity matrix is calculated as follows:

(2) $$\begin{array}{cc} S^{m} & =\frac{\beta_{1}S_{G}^{m}+\beta_{2}S_{D}^{m}}{\sum_{j=1}^{2}\beta_{j}} \end{array}$$

where $S^{m}\in R^{nm\times nm}$ denotes the integrated miRNA similarity matrix, and $\beta_{j}$ indicates the weight of the *j*-th miRNA similarity matrix, which is also set to 1.

### 2.4. SM–miRNA Heterogeneous Network and Target Matrix

In this section, we detail the construction of an SM–miRNA heterogeneous network. First, we built an SM similarity network containing ns SM nodes, in which the similarity scores between SMs were used as the weights of the edges. Then, we constructed a miRNA similarity network with nm miRNA nodes and utilized the similarity scores between miRNAs as the weights of the edges. Finally, we connected these two similarity networks based on known SM–miRNA associations to construct the SM–miRNA heterogeneous network. We consider the adjacency matrix of this heterogeneous network as the target matrix as shown in Formula (3).

(3) $$\begin{array}{cc} H & =\left[\begin{array}{cc} S^{sm} & M\\ M^{T} & S^{m} \end{array}\right] \end{array}$$

where $S^{sm}\in R^{ns\times ns}$, $M\in R^{ns\times nm}$, $M^{T}\in R^{nm\times ns}$, $S^{m}\in R^{nm\times nm}$, $H\in R^{\left(ns+nm)\times (ns+nm\right)}$.

### 2.5. AMCSMMA

#### 2.5.1. Overview

Predicting potential associations between small molecules and miRNAs can be considered as a matrix completion problem, which means recovering the elements with a value of 0 in the association matrix. In this study, we propose a predictive model called AMCSMMA, and its framework is shown in Figure 1. Initially, we introduce and integrate different SM/miRNA similarities. The SM–miRNA heterogeneous network is then constructed, and its adjacency matrix is considered as the target matrix. After that, we design an optimization framework and implement an effective two-step iterative scheme to solve it. Finally, we obtain the prediction score matrix by matrix division.

#### 2.5.2. Optimization Framework

Based on the assumption that the underlying matrix has a low-rank structure, this matrix completion problem is mathematically described in the following form.

(4) $$\begin{array}{cc} \; & \min_{X}\mathrm{rank}\left(X\right)\;\;s.t.\;P_{\Omega }\left(X\right)=P_{\Omega }\left(H\right) \end{array}$$

where $X\in R^{\left(ns+nm)\times (ns+nm\right)}$, rank(·) denotes the rank function, Ω denotes the indices of the observed entries of *H*, and $P_{\Omega }$ is the orthogonal projection operator onto the span of matrices vanishing outside of Ω.

(5) $$\begin{array}{cc} {\left(P_{\Omega }\left(X\right)\right)}_{ij} & =\left\{\begin{array}{cc} X_{ij} & if(i,j)\in \Omega \\ 0 & if(i,j)\notin \Omega \end{array}\right\} \end{array}$$

Unfortunately, owing to the existence of non-convexity and the discontinuous nature in the rank function, the optimization problem (4) becomes an NP-hard problem. Fazel M. [40] proposed a convex relaxation strategy as follows:

(6) $$\begin{array}{c} \min_{X}{∥X∥}_{*}\;\;s.t.\;P_{\Omega }\left(X\right)=P_{\Omega }\left(H\right) \end{array}$$

where ${∥X∥}_{*}=\sum_{i=1}^{ns+nm}\sigma_{i}$ denotes the nuclear norm of *X*, $\sigma_{i}$ is the *i*-th singular value of *X*, which satisfies the relationship of $\sigma_{1}\geq \sigma_{2}\geq \ldots \geq \sigma_{i}\geq \ldots \geq \sigma_{\left(ns+nm\right)}$. With strong theoretical guarantees, the optimization algorithms for nuclear norm regularization frequently achieve the biased solution in practical applications. This occurs because the nuclear norm treats the singular values differently compared to the rank function, in which all the nonzero singular values have equal contributions to the true rank.

Inspired by Hu et al. [41], on the premise that the rank of the underlying matrix is *r* (r<<(ns+nm)), we found that *r* only corresponds to the *r* largest singular values. Therefore, we obtained a more accurate approximation to the rank function, as shown in Formula (7), by minimizing the smallest ns+nm−r singular values and leaving the *r* largest singular values to be free.

(7) $$\begin{array}{c} \min_{X}{∥X∥}_{r}\;\;s.t.\;P_{\Omega }\left(X\right)=P_{\Omega }\left(H\right) \end{array}$$

where ${∥X∥}_{r}=\sum_{i=r+1}^{ns+nm}\sigma_{i}$ denotes the truncated nuclear norm of *X*. Considering that the optimization problem (7) is non-convex, it needs to be rewritten as (8). The complete process of proof can be found in Appendix A.1.

(8) $$\begin{array}{c} \min_{X}{∥X∥}_{*}-\max_{AA^{T}=I,BB^{T}=I}\mathrm{Tr}\left(AXB^{T}\right)\;\;s.t.\;P_{\Omega }\left(X\right)=P_{\Omega }\left(H\right) \end{array}$$

where $A\in R^{r\times (ns+nm)}$, $B\in R^{r\times (ns+nm)}$, and $I\in R^{r\times r}$ denotes the identity matrix. It is necessary to elaborate when $\mathrm{Tr}(AXB^{T})$ obtains the maximum value. The Singular Value Decomposition (SVD) to *X* is as follows:

(9) $$\begin{array}{c} \left(U,\;S,\;V^{T}\right)=SVD\left(X\right) \end{array}$$

where $U=\left(u_{1},\ldots ,u_{r},\ldots ,u_{\left(ns+nm\right)}\right)\in R^{\left(ns+nm)\times (ns+nm\right)}$ and $V=(v_{1},\ldots ,v_{r},\ldots ,v_{\left(ns+nm\right)})$$\in R^{\left(ns+nm)\times (ns+nm\right)}$ are unitary matrices, and $S\in R^{\left(ns+nm)\times (ns+nm\right)}$. Some previous research [41,42] suggested that $\mathrm{Tr}(AXB^{T})$ obtains the maximum value that equals $\sum_{i=1}^{r}\sigma_{i}\left(X\right)$ when *A* equals $U_{r}^{T}\in R^{r\times (ns+nm)}$ and *B* equals $V_{r}^{T}\in R^{r\times (ns+nm)}$, where $U_{r}=\left(u_{1},\ldots ,u_{r}\right)\in R^{(ns+nm)\times r}$ and $V_{r}=\left(v_{1},\ldots ,v_{r}\right)\in R^{(ns+nm)\times r}$. The calculation proof is as follows:

(10) $$\begin{array}{c} \mathrm{Tr}\left(AXB^{T}\right)=\mathrm{Tr}\left({\left(u_{1},u_{2},\ldots ,u_{r}\right)}^{T}USV^{T}\left(v_{1},v_{2},\ldots ,v_{r}\right)\right)=\mathrm{Tr}\left(diag\left(\sigma_{1}\left(X\right),\sigma_{2}\left(X\right),\ldots ,\sigma_{r}\left(X\right),0,\ldots ,0\right)\right)=\sum_{i=1}^{r}\sigma_{i}\left(X\right) \end{array}$$

Further, to avoid the interference of noisy data on the results, we relax the tight constraint as a part of the objective function and set the parameter α to control the weight of this term in the objective function. Additionally, we constrain the completed values between 0 and 1. Ultimately, the optimization framework is described as follows:

(11) $$\begin{array}{c} \min_{X}{∥X∥}_{*}-\max_{AA^{T}=I,BB^{T}=I}\mathrm{Tr}\left(AXB^{T}\right)+\frac{\alpha }{2}{∥P_{\Omega }\left(X\right)-P_{\Omega }\left(H\right)∥}_{F}^{2}\;\;s.t.\;0\leq X_{ij}\leq 1,\;0\leq i,j\leq \left(ns+nm\right) \end{array}$$

where ${∥\cdot ∥}_{F}$ denotes the Frobenius norm.

#### 2.5.3. Optimization Algorithm

In this section, we design an effective two-step iterative algorithm. We initialize $X_{1}$ as $P_{\Omega }\left(H\right)$. In the first step of the *l*-th iteration, we implement the SVD algorithm to $X_{l}$ to obtain the left singular matrix $U_{l}$ and the right singular matrix $V_{l}$. Then, we construct the truncated matrices $A_{l}\;and\;B_{l}$ by, respectively, utilizing the first r-columns of $U_{l}\;and\;V_{l}$. In the second step of the *l*-th iteration, we fix $A_{l},B_{l}$ and update $X_{l+1}$ by solving the following convex sub-problem.

(12) $$\begin{array}{c} X_{l+1}=\underset{Z}{\arg\min}{∥Z∥}_{*}-\mathrm{Tr}\left(A_{l}ZB_{l}^{T}\right)+\frac{\alpha }{2}{∥P_{\Omega }\left(Z\right)-P_{\Omega }\left(H\right)∥}_{F}^{2}\;\;s.t.\;0\leq Z_{ij}\leq 1,\;0\leq i,j\leq \left(ns+nm\right) \end{array}$$

where $Z\in R^{\left(ns+nm)\times (ns+nm\right)}$. According to different datasets, we set the maximal iterations in [1, 4].

For solving the convex sub-problem (12), we introduce the Alternating Direction Multiplier Method (ADMM). Specifically, we introduce the auxiliary matrix *W*, which satisfies Z=W, and the optimization problem is given by (13).

(13) $$\begin{array}{c} \min_{Z}{∥Z∥}_{*}-\mathrm{Tr}\left(A_{l}WB_{l}^{T}\right)+\frac{\alpha }{2}{∥P_{\Omega }\left(W\right)-P_{\Omega }\left(H\right)∥}_{F}^{2}\;\;s.t.\;Z=W,\;0\leq W_{ij}\leq 1,\;0\leq i,j\leq \left(ns+nm\right) \end{array}$$

The Augmented Lagrangian Function of (13) is described as:

(14) $$\begin{array}{cc} \; & L\left(W,Z,Y,\alpha ,\beta \right)={∥Z∥}_{*}-\mathrm{Tr}\left(A_{l}WB_{l}^{T}\right)+\frac{\alpha }{2}{∥P_{\Omega }\left(W\right)-P_{\Omega }\left(H\right)∥}_{F}^{2}+\mathrm{Tr}\left(Y^{T}\left(Z-W\right)\right)+\frac{\beta }{2}{∥Z-W∥}_{F}^{2} \end{array}$$

where *Y* is the Lagrange multiplier, and β>0 is the penalty parameter. We initialize the variables $W_{1},\;Z_{1}\;and\;Y_{1}$ as $P_{\Omega }\left(H\right)$ and update the variables alternately by minimizing the Augmented Lagrange Function L(W,Z,Y,α,β) with respect to the variables in a Gauss–Seidel manner. The exact procedure of the *k*-th iteration is shown below.

**Computing**$W_{k+1}$: Fix $Z_{k},\;Y_{k}$ and minimize the Augmented Lagrangian Function $L(W,Z_{k},Y_{k},\alpha ,\beta )$ for updating $W_{k+1}$.

(15) $$\begin{array}{c} W_{k+1}=\underset{0\leq W_{ij}\leq 1}{\arg\min}{∥Z_{k}∥}_{*}-\mathrm{Tr}\left(A_{l}WB_{l}^{T}\right)+\frac{\alpha }{2}{∥P_{\Omega }\left(W\right)-P_{\Omega }\left(H\right)∥}_{F}^{2}+\mathrm{Tr}\left(Y_{k}^{T}\left(Z_{k}-W\right)\right)+\frac{\beta }{2}{∥Z_{k}-W∥}_{F}^{2} \end{array}$$

Discarding the constant terms, the above equation can be rewritten as (16).

(16) $$\begin{array}{cc} W_{k+1}= & \underset{0\leq W_{\mathrm{ij}}\leq 1}{\arg\min}-\mathrm{Tr}\left(A_{l}WB_{l}^{T}\right)+\frac{\alpha }{2}{∥P_{\Omega }\left(W\right)-P_{\Omega }\left(H\right)∥}_{F}^{2}+\mathrm{Tr}\left(Y_{k}^{T}\left(Z_{k}-W\right)\right)+\frac{\beta }{2}{∥Z_{k}-W∥}_{F}^{2} \end{array}$$

Ignoring the constraint, $L(W,Z_{k},Y_{k},\alpha ,\beta )$ obtains the minimum value when and only when the derivative of (16) equals zero as follows:

(17) $$\begin{array}{cc} -A_{l}^{T}B_{l}+\alpha P_{\Omega }^{*}\left(P_{\Omega }\left({\bar{W}}_{k+1}\right)-P_{\Omega }\left(H\right)\right)-Y_{k}-\beta \left(Z_{k}-{\bar{W}}_{k+1}\right) & =0 \end{array}$$

where $P_{\Omega }^{*}$ denotes the adjoint operator of $P_{\Omega }$ that satisfies $P_{\Omega }^{*}P_{\Omega }=P_{\Omega }$, and ${\bar{W}}_{k+1}$ denotes the transition matrix, which is calculated as Equation (18). The complete calculation process can be found in Appendix A.2.

(18) $$\begin{array}{c} {\bar{W}}_{k+1}=\left(\frac{1}{\beta }Y_{k}+\frac{\alpha }{\beta }P_{\Omega }\left(H\right)+\frac{1}{\beta }A_{l}^{T}B_{l}+Z_{k}\right)-\frac{\alpha }{\alpha +\beta }P_{\Omega }\left(\frac{1}{\beta }Y_{k}+\frac{\alpha }{\beta }P_{\Omega }\left(H\right)+\frac{1}{\beta }A_{l}^{T}B_{l}+Z_{k}\right) \end{array}$$

To update $W_{k+1}$, we implement the operation as Equation (19) on the matrix ${\bar{W}}_{k+1}$, which limits the completed values in [0, 1].

(19) $$\begin{array}{cc} W_{{k+1}_{ij}} & =\left\{\begin{array}{cc} 0 & \mathrm{if}\;{\bar{W}}_{{k+1}_{ij}}\leq 0\\ {\bar{W}}_{{k+1}_{ij}} & \mathrm{if}\;0<{\bar{W}}_{{k+1}_{ij}}<1\\ 1 & \mathrm{if}\;{\bar{W}}_{{k+1}_{ij}}\geq 1 \end{array}\right. \end{array}$$

**Computing**$Z_{k+1}$: Fix $W_{k+1},\;Y_{k}$ and update $Z_{k+1}$ by minimizing $L(W_{k+1},Z,Y_{k},\alpha ,\beta )$.

(20) $$\begin{array}{cc} \; & Z_{k+1}=\underset{Z}{\arg\min}{∥Z∥}_{*}-\mathrm{Tr}\left(A_{l}W_{k+1}B_{l}^{T}\right)+\frac{\alpha }{2}{∥P_{\Omega }\left(W_{k+1}\right)-P_{\Omega }\left(H\right)∥}_{F}^{2}+\mathrm{Tr}\left(Y_{k}^{T}\left(Z-W_{k+1}\right)\right)+\frac{\beta }{2}{∥Z-W_{k+1}∥}_{F}^{2} \end{array}$$

Ignoring the constant terms, we obtain Equation (21).

(21) $$\begin{array}{cc} Z_{k+1} & =\underset{Z}{\arg\min}{∥Z∥}_{*}+\mathrm{Tr}\left(Y_{k}^{T}\left(Z-W_{k+1}\right)\right)+\frac{\beta }{2}{∥Z-W_{k+1}∥}_{F}^{2}\\ \; & =\underset{Z}{\arg\min}{∥Z∥}_{*}+\frac{\beta }{2}{∥Z-\left(W_{k+1}-\frac{1}{\beta }Y_{k}\right)∥}_{F}^{2} \end{array}$$

According to the singular value shrinkage operator $D_{\tau }$ and the related theorem [43], the updating formula is described as follows:

(22) $$\begin{array}{cc} Z_{k+1} & =D_{\frac{1}{\beta }}\left(W_{k+1}-\frac{1}{\beta }Y_{k}\right) \end{array}$$

where $D_{\tau }\left(L\right)=UD_{\tau }\left(S\right)V^{T}$, τ is the threshold parameter, $U,S,V^{T}=SVD\left(L\right)$, $D_{\tau }\left(S\right)=\mathrm{diag}\left(\max\left\{0,\sigma_{i}-\tau \right\}\right)$, $\sigma_{i}$ denotes the main diagonal elements of *S*.

**Computing**$Y_{k+1}$: Fix $W_{k+1}$, $Z_{k+1}$ and update the Lagrange multiplier $Y_{k+1}$ using the gradient ascent method.

(23) $$\begin{array}{cc} Y_{k+1} & =Y_{k}+\gamma \beta \left(\frac{\partial L(W_{k+1},Z_{k+1},Y,\alpha ,\beta )}{\partial Y}\right)\\ & =Y_{k}+\gamma \beta \left(Z_{k+1}-W_{k+1}\right) \end{array}$$

where γ is the learning rate.

Ultimately, we set the iterative stop conditions for the sub-problem according to previous  research [44].

(24) $$\begin{array}{cc} d1_{k+1} & =\frac{{∥Z_{k+1}-Z_{k}∥}_{F}}{{∥Z_{k}∥}_{F}}\leq \varepsilon_{1}\\ d2_{k+1} & =\frac{\left|d1_{k+1}-d1_{k}\right|}{\max\left\{\left|d1_{k}\right|,1\right\}}\leq \varepsilon_{2} \end{array}$$

where $\varepsilon_{1}$ and $\varepsilon_{2}$ are the given accuracies.

After the two-step iterative algorithm converges, we obtain the result matrix and divide it as follows:

(25) $$\begin{array}{cc} H^{\prime } & =\left[\begin{array}{cc} S^{sm^{\prime }} & M^{\prime }\\ {M^{\prime }}^{T} & S^{m^{\prime }} \end{array}\right] \end{array}$$

where $S^{sm^{\prime }}/S^{m^{\prime }}$ is the enhanced SM/miRNA similarity matrix that contains more precise and abundant SM/miRNA similarity information, $M^{\prime }$ is the prediction score matrix, and recovering values are considered as the association scores that represent the possibility of potential association. The complete pseudocode and parameter settings are shown in Algorithm 1.
**Algorithm 1** AMCSMMA.**Require:** *M*, $\left(S_{S}^{sm},\;S_{C}^{sm},\;S_{F}^{sm},\;S_{P}^{sm}\right)\in R^{ns\times ns}$, $\left(S_{G}^{m},\;S_{D}^{m}\right)\in R^{nm\times nm}$, $P_{\Omega }\in R^{\left(ns+nm)\times (ns+nm\right)}$**Ensure:** $M^{\prime }$1:$S^{sm}\in R^{ns\times ns}\leftarrow Matrix\_Fusion\left(S_{S}^{sm},\;S_{C}^{sm},\;S_{F}^{sm},\;S_{P}^{sm},\;\alpha_{i}\right)$2:$S^{m}\in R^{nm\times nm}\leftarrow Matrix\_Fusion\left(S_{G}^{m},\;S_{D}^{m},\;\beta_{i}\right)$3:$H:\left[\begin{array}{cc} S^{sm} & M\\ M^{T} & S^{m} \end{array}\right]$$\in R^{\left(ns+nm)\times (ns+nm\right)}$4:$X_{1}\leftarrow P_{\Omega }\left(H\right)$, l←0, r←1, iterations←3,maxiter←300, $\varepsilon_{1}=2\times 10^{-3}$, $\varepsilon_{2}=10^{-5}$, α=1, β=10, γ=15:**repeat**6:   l←l+1, $\left(U_{l},\;S_{l},\;V_{l}^{T}\right)\leftarrow SVD\left(X_{l}\right)$, where $U_{l}:\left(u_{1},u_{2},\ldots ,u_{r},\ldots ,u_{\left(ns+nm\right)}\right)$, $V_{l}:\left(v_{1},v_{2},\ldots ,v_{r},\ldots ,v_{\left(ns+nm\right)}\right)$7:   $A_{l}\leftarrow {\left(u_{1},u_{2},\ldots ,u_{r}\right)}^{T}$, $B_{l}\leftarrow {\left(v_{1},v_{2},\ldots ,v_{r}\right)}^{T}$8:   $W_{1},\;Z_{1},\;Y_{1}\leftarrow P_{\Omega }\left(H\right)$, k←09:   **repeat**10:     k←k+1,11:     ${\bar{W}}_{k+1}\leftarrow \left(\frac{1}{\beta }Y_{k}+\frac{\alpha }{\beta }P_{\Omega }\left(H\right)+\frac{1}{\beta }A_{l}^{T}B_{l}+Z_{k}\right)-\frac{\alpha }{\alpha +\beta }P_{\Omega }\left(\frac{1}{\beta }Y_{k}+\frac{\alpha }{\beta }P_{\Omega }\left(H\right)+\frac{1}{\beta }A_{l}^{T}B_{l}+Z_{k}\right)$12:     $W_{{k+1}_{ij}}\leftarrow \left\{\begin{array}{cc} 0 & \mathrm{if}\;{\bar{W}}_{{k+1}_{ij}}\leq 0\\ {\bar{W}}_{{k+1}_{ij}} & \mathrm{if}\;0<{\bar{W}}_{{k+1}_{ij}}<1\\ 1 & \mathrm{if}\;{\bar{W}}_{{k+1}_{ij}}\geq 1 \end{array}\right\}$13:     $Z_{k+1}\leftarrow D_{\frac{1}{\beta }}\left(W_{k+1}-\frac{1}{\beta }Y_{k}\right)$, $Y_{k+1}\leftarrow Y_{k}+\gamma \beta \left(Z_{k+1}-W_{k+1}\right)$14:   **until** $\left\{d1_{k+1}\leftarrow \frac{{∥Z_{k+1}-Z_{k}∥}_{F}}{{∥Z_{k}∥}_{F}}\leq \varepsilon_{1}\;and\;d2_{k+1}\leftarrow \frac{\left|d1_{k+1}-d1_{k}\right|}{\max\left\{\left|d1_{k}\right|,1\right\}}\leq \varepsilon_{2}\right\}$ or k==maxiter15:   $X_{l+1}\leftarrow W_{k+1}$16:**until** l==iterations17:$H^{\prime }:\left[\begin{array}{cc} S^{sm^{\prime }} & M^{\prime }\\ {M^{\prime }}^{T} & S^{m^{\prime }} \end{array}\right]$$\in R^{\left(ns+nm)\times (ns+nm\right)}$$\leftarrow X_{l+1}$18:$M^{\prime }\in R^{ns\times nm}\leftarrow Matrix\_Devide\left(H^{\prime }\right)$19:**return** $M^{\prime }$

## 3. Results

### 3.1. Validation Experiment A

In this section, we design Validation Experiment A to quantitatively analyze the effect of the truncated position *r* on the predictive performance of AMCSMMA. Specifically, all confirmed associations in Dataset 1 are regarded as the training samples, all verified associations in Dataset 3 are treated as the testing samples, and all SM–miRNA pairs in Dataset 1 that are neither part of the training set nor the testing set are considered as the candidate samples.

Under specific r∈{1,3,5,…,11,12,…,16,20,25}, we conduct AMCSMMA only utilizing the training samples to recover the SM–miRNA association matrix with missing values. Then, the association scores of the candidate samples and the testing samples are extracted and arranged in descending order to calculate the False Positive Rate (FPR, 1-specificity) and the True Positive Rate (TPR, sensitivity) at a specific threshold. Furthermore, we set the FPR as the abscissa and the TPR as the ordinate and plot the Receiver Operating Characteristic (ROC) curves based on different thresholds.

**Figure 2:**
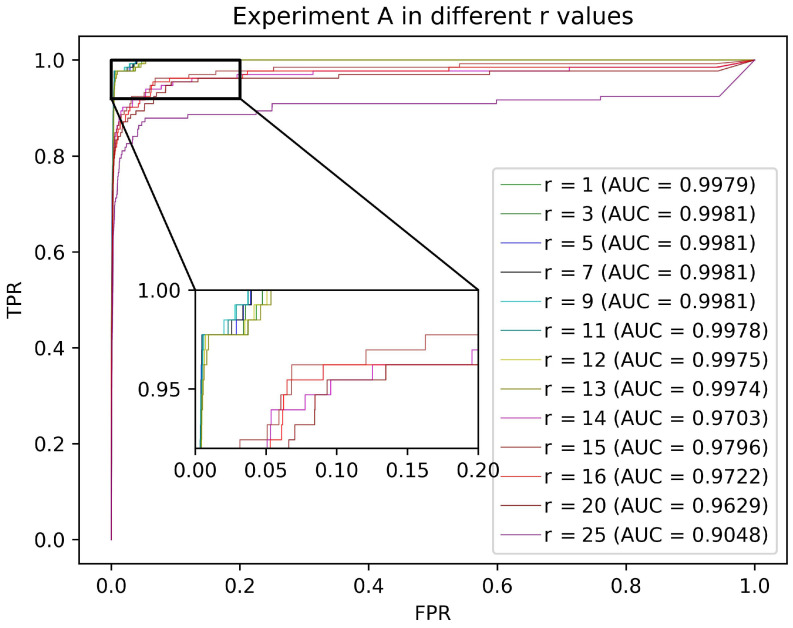
The influence of parameter *r* on the predictive performance of AMCSMMA.

The AUC between 0 and 1 is the area under the ROC curve, and the larger the numerical value is, the better the predictive performance of the model. According to Figure 2, we find that AMCSMMA achieved excellent and stable performance when r∈{1,3,5,…,11,12,13}. With the increase of r value in [1, 13], the computational complexity increased, whereas the prediction accuracy improved weakly. Considering that the adjustable target rank can increase the adaptability of the model to different datasets, we finally set r∈{1,2,3}. The AUC reached 0.9981 at the optimal parameters, which strongly demonstrates the superiority of our model in predicting potential SM–miRNA associations.

### 3.2. Validation Experiment B

To comprehensively evaluate the predictive performance of our model, we design Validation Experiment B, in which 664 confirmed SM–miRNA associations in Dataset 1 are utilized as the training samples, and 132 verified SM–miRNA associations in Dataset 3 are assembled into the positive testing set. Then, we randomly select 132 unknown SM–miRNA associations from Dataset 1 to form the negative testing set. The intersection set of the training set, the positive testing set, and the negative testing set is an empty set.

**Figure 3:**
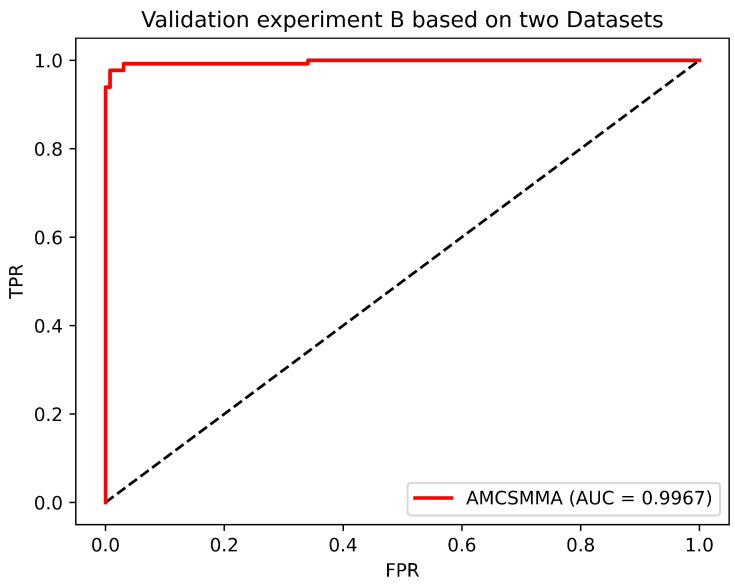
The ROC curve and AUC value of Validation Experiment B.

In addition, the AUC, five additional metrics are introduced, which include the Precision, Recall, F1 Score, Accuracy, and MCC. We calculate the above metrics based on three thresholds that maximize the F1 Score, the Accuracy, and the MCC. Considering the fluctuation of experimental results caused by randomly selecting the negative testing samples, we repeat the above procedure 100 times and consider the average value as the final result. It can be seen from Table 3 and Figure 3 that all metrics achieved a significant result.

### 3.3. Four Cross-Validation Experiments

Based on Dataset 1 and Dataset 2 separately, we implemented five-fold cross-validation (CV), Global Leave-One-Out CV (LOOCV), miRNA-Fixed Local LOOCV, and SM-Fixed Local LOOCV to further validate the predictive performance of AMCSMMA. At the same time, we likewise applied the above four CVs to other association predictive models.

In the five-fold CV, all the confirmed SM–miRNA associations (664 items) were randomly divided into five parts, of which one part incorporated 132 items and each remaining part included 133 items. Specifically, we alternately utilized one part as the testing set, and the remaining four parts were fused as the training set. Additionally, all the unknown SM–miRNA associations were assembled in the candidate set. In each fold, only utilizing the training samples, we conducted AMCSMMA to recover the SM–miRNA association matrix. Likewise as in Validation Experiment A, the association scores of the testing and candidate samples were integrated into a descending sequence.

Then, we plotted the ROC curve and derived the AUC value under this fold. After five folds, the average AUC value was regarded as the result of one five-fold CV. It is worth noting that we repeated the five-fold CV 100 times and took the average AUC value as the final result, which insulates the validation result against the randomness of sample partitioning. Additionally, we calculated the Standard Deviation (SD) value that can reflect the robustness of the model. Finally, the AUC±SD of AMCSMMA under five-fold CV reached 0.9910 ± 0.0004 and 0.8768 ± 0.0039 based on Datasets 1 and 2, respectively.

**Figure 4:**
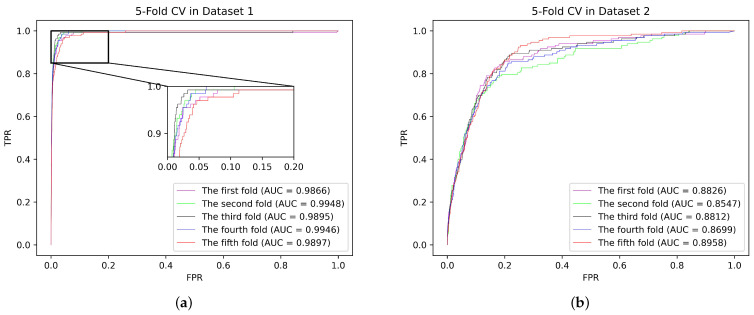
(**a**) The ROC curves and AUC values of five folds based on Dataset 1. (**b**) The ROC curves and AUC values of five folds based on Dataset 2.

From Table 4, we observe that AMCSMMA achieved a higher AUC and a lower SD than did the compared models based on both datasets, which indicates that it has superior predictive performance and robustness. Figure 4 shows the ROC curves of each fold in one five-fold CV based on two datasets and the areas under the curves.

In Global LOOCV, each verified SM–miRNA association was sequentially selected as the testing sample, and the remaining 663 confirmed associations were considered as the training samples. Additionally, all unknown SM–miRNA associations were treated as the candidate samples.

Similarly, we calculated the AUC values successively under 664 folds according to the association scores of the testing and candidate samples and regarded the average AUC as the result. From Table 4, we discover that the AUC of AMCSMMA under Global LOOCV reached 0.9923 (0.8861) based on Dataset 1 (Dataset 2), which exceeds all other models proposed in recent years and once again demonstrates the superior predictive performance of our model.

In miRNA-Fixed Local LOOCV and SM-Fixed Local LOOCV, the testing and training samples were selected in the same way as in Global LOOCV. Nevertheless, the candidate set in miRNA/SM-Fixed Local LOOCV only consisted of the unknown SM–miRNA associations that have the same miRNA/SM with the testing sample in each fold. After several computational steps, the AUC of AMCSMMA reached 0.9898 (0.8880) based on Dataset 1 (Dataset 2) under miRNA-Fixed Local LOOCV, which surpasses all comparative models. The AUC reached 0.8222 (0.7232) based on Dataset 1 (Dataset 2) under SM-Fixed Local LOOCV, which is superior to the other four models (TLHNSMMA, GISMMA, SLHGISMMA, and SMiR-NBI). The DCMF achieved the best performance because it was able to obtain the exact SM feature matrix.

As shown in Table 4, AMCSMMA achieved better performance based on Dataset 1 than on Dataset 2 in cross-validation experiments. The reason for this is that Datasets 1 and 2 provide the same positive samples (divided into training and testing samples), but Dataset 1 provides a much larger number of candidate samples compared with Dataset 2. Since these additional candidate samples contain SMs/miRNAs that have no known associations with miRNAs/SMs, they have relatively low association scores compared to the testing samples, resulting in a higher AUC value based on Dataset 1 than on Dataset 2. Therefore, we expect that the accuracy of AMCSMMA will improve as more SMs and miRNAs are added to the dataset.

### 3.4. Case Studies

#### 3.4.1. The First Type of Case Study

In this section, we initially utilize AMCSMMA to obtain the predictive scores of all unknown SM–miRNA associations in Dataset 1. Subsequently, we count the number of associations confirmed by published literature in PubMed. Finally, 9 (33) among the top 20 (100) associations can be confirmed. Table 5 lists the top 20 associations and the literature evidence (PubMed ID).

Specifically, Khorrami et al. [45] identified that miR-146a is overexpressed in a colon cancer cell line (HT-29), which can increase its resistance to 5-FU and irinotecan, thereby diminishing the prognostic effect of chemotherapy. Additionally, Zhang et al. [46] revealed that CYP11A1 and CYP19A1 expression in human CCs, and the resulting production of progesterone and estradiol, are transcriptionally down-regulated by miR-320a deficiency. Moreover, the colorectal cancer hallmark (CXCL12) is able to induce miR-125 upregulation and generate the chemotherapy drugs 5-FU resistance [47].

We implement this type of case study on other comparative models. From Table 6, our model achieves the best performance.

#### 3.4.2. The Second Type of Case Study

To explore the applicability of AMCSMMA to new SMs, we conducted the second type of case study to two SMs, 5-FU and 5-Aza-2’-deoxycytidine based on Dataset 1. In detail, we first removed all verified associations related to the specific SM. Then, a descending sequence consisting of association scores between the specific SM and all miRNAs was obtained. We counted the number of associations confirmed by the SM2miR database [27] and published references. Finally, in the second type to 5-FU, 20 (34) among the top 20 (50) associations were confirmed as shown in Table 7.

Specifically, the sensitivity of 5-FU was significantly correlated with the antitumor effect, and overexpression of miR-329 and let-7c enhanced the sensitivity of 5-FU by affecting the apoptotic pathway, thus enhancing the antitumor effect [48,49]. In another study, Wang et al. [50] found that 5-FU was abnormally sensitive to MCF-7 cells due to its negative regulation on Bcl-xl expression via let-7b. Additionally, Bamodu et al. [51] concluded that the SOD2-enhanced 5-FU chemoresistance of colorectal cancer cells was inhibited by inducing the re-expression of hsa-miR-324. Furthermore, Han et al. [52] discovered that miR-874 can reduce the resistance of colorectal cancer cells to 5-FU.

In the second type to 5-Aza-2’-deoxycytidine, 16 (26) of the top 20 (50) associations were confirmed as shown in Table 8. Particularly, Liu et al. [53] found that the demethylation agent 5-Aza-2’-deoxycytidine inhibited the proliferation of esophageal cancer cells by increasing the expression of miR-203a. Moreover, the expression of miR-19b and let-7b increased in gastric cancer cells after 5-Aza-2’-deoxycytidine treatment [54,55]. In addition, Sun et al. [56] found that hypermethylation of the promoter region in gastrointestinal cancer cell lines correlated with the expression of miR-148a in gastric cancer, and thus treatment with the demethylation agent 5-Aza-2’-deoxycytidine can be performed.

Furthermore, we conducted the second type of case study on BNNRSMMA and DCMF, which are both heuristic algorithms based on matrix completion. As shown in Table 9, our model achieved the best performance except in Number D.

In conclusion, the above experimental results demonstrate that AMCSMMA is an excellent model with superior predictive performance and high robustness in predicting potential SM–miRNA associations, which can provide guidance for complex and expensive biological experiments and accelerate the discovery of new SM–miRNA associations, thus facilitating drug development and disease treatment.

## 4. Discussion

In recent years, an increasing number of studies have shown that the abnormal expression of miRNAs is closely related to a variety of physiological and pathological processes, including cancer, cardiovascular diseases, and metabolic diseases [13,57]. As a result, targeting and modulating miRNAs with small molecule (SM) drugs has become a significant modality for clinical treatment.

Given the complexity and expense of developing new SMs, it is extremely difficult to develop specific SMs for each dysregulated miRNA. Therefore, exploring potential associations between known SMs and miRNAs is both significant and urgent in drug development and disease treatment. Since confirming SM–miRNA associations through biological experiments is time-consuming and expensive, more effective predictive approaches need to be proposed for identifying the SM–miRNA associations with high association probabilities, which can provide guidance for biological experiments and discover potential SM–miRNA associations more cost-effectively.

In this paper, we proposed a more accurate predictive model based on the truncated nuclear norm, called AMCSMMA. After determining the optimal parameter values, the results of Validation Experiment A, four cross-validation experiments, Validation Experiment B, and two types of case studies indicated that AMCSMMA had superior prediction accuracy and high robustness. The reasons for this are discussed in the following.

- All the known SM–miRNA associations were acquired from the SM2miR v1.0 database [27] and the published experimental literature, which are extremely reliable.
- We constructed the SM–miRNA heterogeneous network and defined its adjacency matrix as the target matrix. This not only well utilized similarity information but also enriched it as the iteration progressed.
- Unlike the nuclear norm regularization, the truncated nuclear norm regularization only minimized the sum of partial singular values, which not only made the result matrix more closely approximate the true solution but also improved the adaptability to different datasets.
- We designed an effective two-step iterative scheme to solve the optimization problem.

Although the advancement of AMCSMMA in predicting potential SM–miRNA associations enables it to provide reliable guidance for biological experiments, the model still has some limitations. For instance, the small number of known SM–miRNA associations greatly restricts the prediction accuracy of our model. Moreover, the biological data closely related to SM or miRNA, such as lncRNA and the circRNA, can be introduced to construct heterogeneous networks with more information to improve the prediction accuracy. Furthermore, the work of Yu et al. [58] inspired the idea that deep-learning-based approaches may be able to achieve good results. Due to the multiple utilization of the SVD algorithm, our model requires a relatively high time cost, which will be the focus of our future research.

## Figures and Tables

**Table 1:** The main innovative points and limitations of the proposed models.

Model	Main Innovative Points	Main Limitations
BNNRSMMA	Bounded nuclear norm	Failing to obtain the unbiased solution and adjust the target rank
DCMF	WKNKN method	Failing to adjust the target rank
TLHNSMMA	Triple layer heterogeneous network	Failing to predict miRNAs/SMs associated with new SMs/miRNAs
GISMMA	Graphlet interactions	Failing to avoid noise interference
SLHGISMMA	Sparse learning method (SLM)	Failing to restrict prediction scores in [0, 1]
SMiR-NBI	Resources allocation	Failing to predict miRNAs/SMs associated with new SMs/miRNAs

**Table 2:** The complete data information for three datasets.

Dataset	Number of SMs: ns	Number of miRNAs: nm	Number of Known Associations	Number of Unknown Associations	Dimension of Association Matrix
Dataset 1	831	541	664	448,907	831×541
Dataset 2	39	286	664	10,490	39×286
Dataset 3	831	541	132	449,439	831×541

**Table 3:** The result of Experiment B in terms of the Precision, Recall, F1 Score, Accuracy, and MCC value.

Threshold Setting	Precision	Recall	F1 Score	Accuracy	MCC
$t_{1}$	0.9851	0.9861	0.9856	0.9855	0.9711
$t_{2}$	0.9882	0.9830	0.9855	0.9856	0.9708
$t_{3}$	0.9868	0.9839	0.9852	0.9853	0.9713

Note: $t_{1},\;t_{2},$ and $\;t_{3}$ are the thresholds that maximize the F1 Score, Accuracy, and MCC separately.

**Table 4:** The result comparison in terms of the AUC values between AMCSMMA, BNNRSMMA, DCMF, TLHNSMMA, GISMMA, SLHGISMMA, and SMiR-NBI in four kinds of cross-validation experiments based on two datasets.

Dataset	Model	5-Fold CV	Global LOOCV	miRNA-Fixed Local LOOCV	SM-Fixed Local LOOCV
Dataset1	AMCSMMA	0.9910±0.0004	0.9923	0.9898	0.8222
	BNNRSMMA	0.9758 ± 0.0029	0.9822	0.9793	0.8253
	DCMF	0.9836 ± 0.0030	0.9868	0.9833	**0.8377**
	TLHNSMMA	0.9851 ± 0.0012	0.9859	0.9845	0.7645
	GISMMA	0.9263 ± 0.0026	0.9291	0.9505	0.7702
	SLHGISMMA	0.9241 ± 0.0052	0.9273	0.9365	0.7703
	SMiR-NBI	0.8554 ± 0.0063	0.8843	0.8837	0.7497
Dataset2	AMCSMMA	0.8768±0.0039	0.8861	0.8880	0.7232
	BNNRSMMA	0.8759 ± 0.0041	0.8433	0.8852	0.7350
	DCMF	0.8632 ± 0.0042	0.8770	0.8836	**0.7591**
	TLHNSMMA	0.8168 ± 0.0022	0.8149	0.8244	0.6057
	GISMMA	0.8088 ± 0.0044	0.8203	0.8640	0.6591
	SLHGISMMA	0.7724 ± 0.0032	0.7774	0.7973	0.6556
	SMiR-NBI	0.7104 ± 0.0087	0.7264	0.7846	0.6100

Note: Each bold value means that it is the best value in the experiment.

**Table 5:** The top 20 SM–miRNA associations predicted by AMCSMMA in the first type of case study.

Small Molecule	miRNA	Evidence	Small Molecule	miRNA	Evidence
CID:3385	hsa-mir-125b-1	28176874	CID:3385	hsa-let-7b	25789066
CID:3385	hsa-mir-125b-2	28176874	CID:3385	hsa-mir-126	26062749
CID:36314	hsa-mir-518c	unconfirmed	CID:3385	hsa-mir-26a-2	unconfirmed
CID:3385	hsa-mir-26a-1	unconfirmed	CID:3229	hsa-let-7g	unconfirmed
CID:3385	hsa-mir-107	26636340	CID:3385	hsa-mir-181b-1	19948396
CID:3229	hsa-let-7e	unconfirmed	CID:3385	hsa-mir-146a	28466779
CID:3385	hsa-mir-103a-1	unconfirmed	CID:451668	hsa-mir-15b	unconfirmed
CID:3229	hsa-mir-27b	unconfirmed	CID:60750	hsa-mir-23a	unconfirmed
CID:451668	hsa-mir-23a	unconfirmed	CID:3229	hsa-mir-27a	unconfirmed
CID:3385	hsa-mir-181a-1	29795190	CID:3385	hsa-mir-155	28347920

Note: (1) The top 1–10 associations and corresponding evidence are presented in the first three columns, while the top 11–20 are presented in the last three columns. (2) CID denotes the compound number from the Pubchem database. (3) Evidence shows the PubMed IDs of the experimental literature.

**Table 6:** The number of confirmed SM–miRNA associations in the top 20 associations predicted by AMCSMMA and other models separately.

	AMCSMMA	BNNRSMMA	DCMF	TLHNSMMA	GISMMA	SLHGISMMA
Number	9	6	7	7	1	5

**Table 7:** The top 50 SM–miRNA associations predicted by AMCSMMA in the second type of case study to the SM 5-FU.

miRNA	Evidence	miRNA	Evidence
hsa-let-7a-1	26198104	hsa-mir-217	unconfirmed
hsa-let-7b	25789066	hsa-mir-23a	26198104
hsa-let-7c	25951903	hsa-mir-24-2	26198104
hsa-let-7d	26198104	hsa-mir-26a-1	unconfirmed
hsa-mir-1226	26198104	hsa-mir-27a	26198104
hsa-mir-125b-1	28176874	hsa-mir-299	unconfirmed
hsa-mir-125b-2	28176874	hsa-mir-320a	26198104
hsa-mir-128-1	26198104	hsa-mir-324	30103475
hsa-mir-128-2	26198104	hsa-mir-328	unconfirmed
hsa-mir-132	26198104	hsa-mir-329-1	30127965
hsa-mir-133a-1	26198104	hsa-mir-329-2	30127965
hsa-mir-139	27173050	hsa-mir-342	26198104
hsa-mir-155	28347920	hsa-mir-345	unconfirmed
hsa-mir-16-1	26198104	hsa-mir-346	unconfirmed
hsa-mir-18a	26198104	hsa-mir-34b	unconfirmed
hsa-mir-181a-1	29795190	hsa-mir-372	unconfirmed
hsa-mir-181a-2	24462870	hsa-mir-409	unconfirmed
hsa-mir-181b-1	19948396	hsa-mir-412	unconfirmed
hsa-mir-181b-2	19948396	hsa-mir-431	unconfirmed
hsa-mir-24-1	26198104	hsa-mir-455	21743970
hsa-mir-197	26198104	hsa-mir-500a	unconfirmed
hsa-mir-199a-2	26198104	hsa-mir-501	26198104
hsa-mir-202	unconfirmed	hsa-mir-518c	unconfirmed
hsa-mir-21	26198104	hsa-mir-650	unconfirmed
hsa-mir-212	unconfirmed	hsa-mir-874	27221209

Note: (1) Evidence shows the PubMed IDs of the experimental literature. (2) “26198104" denotes the SM2miR v1.0 database [27]. (3) 20 (34) of the top 20 (50) associations were verified successfully.

**Table 8:** The top 50 SM–miRNA associations predicted by AMCSMMA in the second type of case study to the SM 5-Aza-2’-deoxycytidine.

miRNA	Evidence	miRNA	Evidence
hsa-mir-125b-1	26198104	hsa-mir-197	unconfirmed
hsa-mir-125b-2	26198104	hsa-mir-199a-2	unconfirmed
hsa-mir-203a	26577858	hsa-mir-133a-1	unconfirmed
hsa-let-7b	26708866	hsa-mir-133a-2	unconfirmed
hsa-let-7c	24704393	hsa-mir-20a	26198104
hsa-let-7d	26802971	hsa-mir-200c	23626803
hsa-mir-19b-1	25270964	hsa-let-7a-1	unconfirmed
hsa-mir-132	unconfirmed	hsa-mir-205	unconfirmed
hsa-mir-181a-1	26198104	hsa-mir-21	26198104
hsa-mir-181a-2	26198104	hsa-mir-221	unconfirmed
hsa-mir-137	23200812	hsa-mir-222	unconfirmed
hsa-mir-141	unconfirmed	hsa-mir-23a	25213664
hsa-mir-145	26198104	hsa-mir-24-2	26198104
hsa-mir-148a	24920927	hsa-mir-26a-1	unconfirmed
hsa-mir-149	unconfirmed	hsa-mir-27a	26198104
hsa-mir-155	26198104	hsa-mir-27b	26198104
hsa-mir-16-1	26198104	hsa-mir-29a	26198104
hsa-mir-17	26198104	hsa-mir-324	unconfirmed
hsa-mir-18a	unconfirmed	hsa-mir-328	23991164
hsa-mir-19a	26198104	hsa-mir-342	unconfirmed
hsa-mir-1226	unconfirmed	hsa-mir-346	unconfirmed
hsa-mir-181b-1	unconfirmed	hsa-mir-500a	unconfirmed
hsa-mir-181b-2	unconfirmed	hsa-mir-501	unconfirmed
hsa-mir-24-1	26198104	hsa-mir-650	unconfirmed
hsa-mir-194-1	unconfirmed	hsa-mir-874	unconfirmed

Note: (1) Evidence shows the PubMed IDs of the experimental literature. (2) “26198104" denotes the SM2miR v1.0 database [27]. (3) 16 (26) of the top 20 (50) associations were verified successfully.

**Table 9:** The number of confirmed SM–miRNA associations in the top 20/50 associations predicted by AMCSMMA, BNNRSMMA, and DCMF.

Model	Number A	Number B	Number C	Number D
AMCSMMA	20	34	16	26
BNNRSMMA	17	32	16	27
DCMF	17	29	16	27

Note: Number A/B denotes the number of confirmed SM–miRNA associations in the top 20/50 associations to SM 5-FU. Number C/D denotes the number of confirmed SM–miRNA associations in the top 20/50 associations to SM 5-Aza-2’-deoxycytidine.

## Data Availability

The Python code and datasets of AMCSMMA are publicly available at https://github.com/a1657884486/AMCSMMA.git, accessed on 1 April 2023.

## Supplementary Materials

The following supporting information can be downloaded at: https://www.mdpi.com/article/10.3390/cells12081123/s1, Table S1: The known SM–miRNA associations and the relevant experimental literature in Dataset 3.

## Abbreviations

The following abbreviations are used in this manuscript:

SM	Small Molecule
miRNA	microRNA
CMF	Collective Matrix Factorization
CV	Cross-Validation
LOOCV	Leave-One-Out Cross-Validation
MCC	Matthews Correlation Coefficient
SVD	Singular Value Decomposition
ADMM	Alternating Direction Multiplier Method
NP-hard	Non-deterministic Polynomial-hard

## Appendix A

### Appendix A.1

**Proof** **of** **Formula** **(8).** By Von Neumann’s trace inequality, we have:

(A1) $$\begin{array}{c} \mathrm{Tr}\left(AXB^{T}\right)=\mathrm{Tr}\left(XB^{T}A\right)\leq \sum_{i=1}^{ns+nm}\sigma_{i}\left(X\right)\sigma_{i}\left(B^{T}A\right) \end{array}$$

where $X\in R^{\left(ns+nm)\times (ns+nm\right)},\;A\in R^{r\times (ns+nm)}$, $B\in R^{r\times (ns+nm)}$, $AA^{T}=I,\;BB^{T}=I$, r(r≤(ns+nm)) is a non-negative integer, $I\in R^{r\times r}$ denotes the identity matrix, and $\sigma_{i}$ is the *i*-th singular value and satisfies the relationship of $\sigma_{1}\geq \sigma_{2}\geq \ldots \geq \sigma_{i}\geq \ldots \geq \sigma_{\left(ns+nm\right)}\geq 0$. As rank(A)=rank(B)=r, we have $rank(B^{T}A)=s\leq r$. Owing to $B^{T}A{\left(B^{T}A\right)}^{T}=I$, $\sigma_{i}\left(B^{T}A\right)$ equals 1 if i≤s, otherwise it equals 0, and then we have:

(A2) $$\begin{array}{c} \sum_{i=1}^{ns+nm}\sigma_{i}\left(X\right)\sigma_{i}\left(B^{T}A\right)=\sum_{i=1}^{s}\sigma_{i}\left(X\right)\sigma_{i}\left(B^{T}A\right)+\sum_{i=s+1}^{ns+nm}\sigma_{i}\left(X\right)\sigma_{i}\left(B^{T}A\right)=\sum_{i=1}^{s}\sigma_{i}\left(X\right)\cdot 1+\sum_{i=s+1}^{ns+nm}\sigma_{i}\left(X\right)\cdot 0=\sum_{i=1}^{s}\sigma_{i}\left(X\right) \end{array}$$

Combining inequalities A1 and A2, we have:

(A3) $$\begin{array}{c} \mathrm{Tr}\left(AXB^{T}\right)\leq \sum_{i=1}^{ns+nm}\sigma_{i}\left(X\right)\sigma_{i}\left(B^{T}A\right)=\sum_{i=1}^{s}\sigma_{i}\left(X\right)\leq \sum_{i=1}^{r}\sigma_{i}\left(X\right) \end{array}$$

Then, we have:

(A4) $$\begin{array}{c} {\left||X|\right|}_{*}-\max_{AA^{T}=I,BB^{T}=I}\mathrm{Tr}\left(AXB^{T}\right)={\left||X|\right|}_{*}-\sum_{i=1}^{r}\sigma_{i}\left(X\right)={\left||X|\right|}_{r} \end{array}$$

☐

### Appendix A.2

According to the equation ${\left(I+\frac{\alpha }{\beta }P_{\Omega }^{*}P_{\Omega }\right)}^{-1}=\left(I-\frac{\alpha }{\alpha +\beta }P_{\Omega }^{*}P_{\Omega }\right)$ [59], where ${\left(\cdot \right)}^{-1}$ denotes the reverse operator, we have:

(A5) $$\begin{array}{cc} {\bar{W}}_{k+1} & ={\left(I+\frac{\alpha }{\beta }P_{\Omega }^{*}P_{\Omega }\right)}^{-1}\left(\frac{A_{l}^{T}B_{l}}{\beta }+\frac{\alpha }{\beta }P_{\Omega }^{*}P_{\Omega }\left(H\right)+\frac{Y_{k}}{\beta }+Z_{k}\right)\\ & =\left(I-\frac{\alpha }{\alpha +\beta }P_{\Omega }^{*}P_{\Omega }\right)\left(\frac{A_{l}^{T}B_{l}}{\beta }+\frac{\alpha }{\beta }P_{\Omega }^{*}P_{\Omega }\left(H\right)+\frac{Y_{k}}{\beta }+Z_{k}\right)\\ & =\left(\frac{1}{\beta }Y_{k}+\frac{\alpha }{\beta }P_{\Omega }\left(H\right)+\frac{1}{\beta }A_{l}^{T}B_{l}+Z_{k}\right)-\frac{\alpha }{\alpha +\beta }P_{\Omega }\left(\frac{1}{\beta }Y_{k}+\frac{\alpha }{\beta }P_{\Omega }\left(H\right)+\frac{1}{\beta }A_{l}^{T}B_{l}+Z_{k}\right) \end{array}$$
